# FGF Signalling in the Self-Renewal of Colon Cancer Organoids

**DOI:** 10.1038/s41598-019-53907-7

**Published:** 2019-11-22

**Authors:** Jörg Otte, Levent Dizdar, Bianca Behrens, Wolfgang Goering, Wolfram T. Knoefel, Wasco Wruck, Nikolas H. Stoecklein, James Adjaye

**Affiliations:** 1 Institute for Stem Cell Research and Regenerative Medicine, University Hospital and Medical Faculty of the Heinrich-Heine University Düsseldorf, Düsseldorf, Germany; 2 General, Visceral and Paediatric Surgery, University Hospital and Medical Faculty of the Heinrich-Heine University Düsseldorf, Düsseldorf, Germany; 3 Institute for Pathology, University Hospital and Medical Faculty of the Heinrich-Heine University Düsseldorf, Düsseldorf, Germany

**Keywords:** Cancer models, Cancer microenvironment, Cancer stem cells, Colon cancer, Tumour heterogeneity

## Abstract

The progression of colorectal cancer (CRC) is supposedly driven by cancer stem cells (CSC) which are able to self-renew and simultaneously fuel bulk tumour mass with highly proliferative and differentiated tumour cells. However, the CSC-phenotype in CRC is unstable and dependent on environmental cues. Fibroblast growth factor 2 (FGF2) is essential and necessary for the maintenance of self-renewal in adult and embryonic stem cells. Investigating its role in self-renewal in advanced CRC patient-derived organoids, we unveiled that FGF-receptor (FGFR) inhibition prevents organoid formation in very early expanding cells but induces cyst formation when applied to pre-established organoids. Comprehensive transcriptome analyses revealed that the induction of the transcription factor activator-protein-1 (AP-1) together with MAPK activation was most prominent after FGFR-inhibition. These effects resemble mechanisms of an acquired resistance against other described tyrosine kinase inhibitors such as EGF-receptor targeted therapies. Furthermore, we detected elevated expression levels of several self-renewal and stemness-associated genes in organoid cultures with active FGF2 signalling. The combined data assume that CSCs are a heterogeneous population while self-renewal is a common feature regulated by distinct but converging pathways. Finally, we highlight FGF2 signalling as one of numerous components of the complex regulation of stemness in cancer.

## Introduction

In today’s view of tumour biology, cancer stem cells (CSC) are a subpopulation of malignant cells able to self-renew and to serve as an ongoing source for differentiated tumour cells. With their stem cell-like properties, they supposedly play a major role in therapeutic resistance and the metastatic process. However, the CSC-phenotype is an unstable feature dictated by environmental cues^1^. Preserving the tumour’s cellular hierarchy *in vitro*, organoid cell culture systems have progressed to a versatile model with numerous applications such as the analysis of driver mutations after gene modifications or complex drug screens^2^. In colorectal cancer (CRC), stem cell niche requirements seem to diminish with cancer progression and increasing malignancy. CSCs develop a more autonomous phenotype by acquiring gene mutations affecting self-renewal associated pathways such as the Wnt/β-catenin, Transforming growth factor beta (TGF-β), Epidermal growth factor (EGF) or mitogen-activated protein kinase (MAPK)-pathways^1,3–5^.

For some adult stem cells and especially in human pluripotent stem cells, fibroblast growth factor 2 (FGF2) is an important cell culture component essential for the maintenance of self-renewal. In pluripotent stem cells, both induced pluripotent stem cells (iPSCs) and embryonic stem cells (ESCs), FGF2 co-ordinates the expression of several TGF-β family members thus inducing self-renewal via SMAD2/3 phosphorylation while bone morphogenetic proteins (BMPs) and the associated SMAD1/5/8 phosphorylation are suppressed^6^. These factors are not restricted to pluripotent cells, but also play a major role in intestinal homeostasis with BMPs being one of the major inhibitors of intestinal stem cell self-renewal^7^. The influence of the TGF-β pathway is highly context-dependent. In epithelial cells, TGF-β can have tumour suppressive and differentiating effects on healthy or low-malignant cells, whereas it can also promote tumour growth and invasion in mutated cells, which lack the suppressive response^8^. TGF-β can further influence the number of CSCs by stromal expression^9^. In the healthy intestine, FGF2 is important during development, crypt homeostasis and tissue repair after injury. In rat models, FGF2 increases intestinal stem cell survival after radiation^10^.

The aim of our study was to investigate the role of FGF2 in the self-renewal of highly malignant CRC. With our organoid culture model, we selected for most autonomous CSCs with low niche requirements and analysed the effect of FGFR inhibition on organoid formation in these highly malignant cells^1,3,11–13^.

## Results

### FGFR inhibition prevents organoid formation in primary colorectal cancer cells

**Figure 1:**
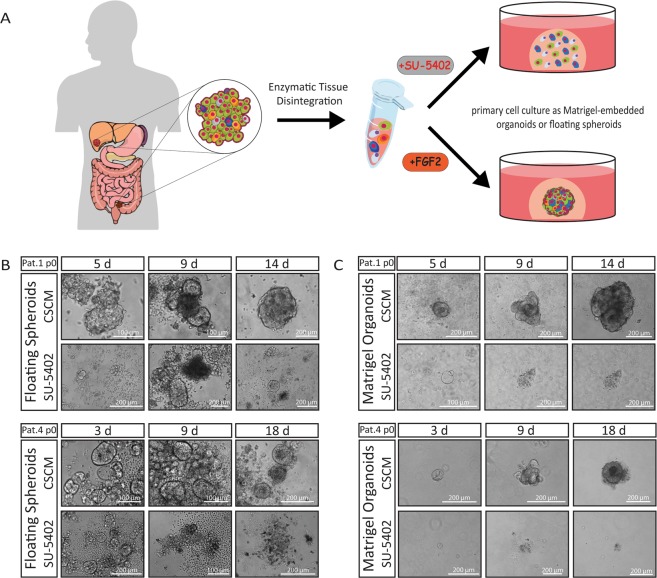
Disabled spheroid and organoid formation by FGFR-inhibition in patient-derived CRC cells. (**A**) Surgical biopsies were enzymatically digested to obtain a suspension of single cells. The naïve unsorted cells were cultured in FGF2 containing CSC-medium or with the FGFR-inhibitor SU-5402. (Parts of the scheme were created in the Mind the Graph platform, www.mindthegraph.com (2019), adaptations were made, Illustrations were made under a creative commons license (https://creativecommons.org/licenses/by-sa/4.0/) (**B**) Cell culture initiation of naïve unsorted bulk cells (p0) after tissue disintegration, cultured in CSC-media (CSCM) or with FGFR-inhibitor (SU-5402) under low-attachment conditions as floating spheres or (**C**) as Matrigel-embedded organoids.

In stem cell culture, serum replacement is crucial for avoiding unwanted differentiation. To select for highly malignant CSCs, which are reported to be non-dependent on exogenous growth factor stimuli such as Wnt, TGF-β or p38-MAPK modulation, our CSC-medium only contains the growth factors EGF and FGF2^12^. We obtained surgical specimens from nine CRC patients and initiated spheroid and organoid cultures from single cell suspensions of naïve unsorted bulk cells without any foregoing selection (Table 1, Fig. 1A). As expected, only biopsies from progressed cancers (with distant metastasis or poorly differentiated) grew in our culture conditions. To analyse the role of FGF2, a major component essential for the self-renewal of pluripotent stem cells, we omitted cytokines from our CSC-medium and added a FGFR-inhibitor, the small molecule SU-5402^14^. To our surprise, we observed that long-term organoid or spheroid culture could not be established under FGFR-inhibition. When we cultured single cell suspensions under low-attachment conditions, cells formed initial spheroids within five days. In Patient 1, we observed a luminal organization reminiscent of an adenoma-like cyto-architecture within 14 d of FGF2-containing CSC-medium culture. Under FGFR-inhibition, however, we detected initial spheroid formation, but also observed morphological differences such as a flattened structure without luminal organization. These initial spheroids stopped proliferating after a few weeks and could not be passaged further. We obtained similar results in spheroids obtained from Patient 4, where initially formed spheroids stopped growing after 9 d under FGFR-inhibition (Fig. 1B).

**Table 1:** Clinical data.

ID	Gender	Age	Location sampled tissue	UICC	Primary Tumour (TNM)	Differentiation	Pre-treatment	BRAF/NRAS/KRAS
Patient 1	M	62	Liver	IV	pT2 pN1 M1hep	Moderate	5-FU, Oxaliplatin, Bevacizumab	wt/wt/G12D
Patient 2	M	64	para rectal LN	IV	pT2 pN2 M1hep	Moderate	5-FU, Oxaliplatin, Cetuximab	wt/wt/wt
Patient 3	F	81	Sigmoid	IV	pT3 pN1 M1hep	Moderate	/	wt/G12V/wt
Patient 4	M	51	Sigmoid	IB	pT2 pN0 M0	Poor	/	wt/G12S/wt
Patient 5	F	59	Rectum	IB	pT2 pN0 M0	Well	Capecitabine, Radiotherapy (50,4 Gy)	
Patient 6	F	58	Rectum		HGIEN		/	
Patient 7	F	85	Rectum	IIA	pT3 pN0 M0	Well	/	
Patient 8	M	74	Liver	IV	pT2 pN0 M1hep	Moderate	/	
Patient 9	M	75	Sigmoid	IIA	pT3 pN0 M0	Well	/	

Clinical data of sampled patients. Organoid cultures could be established from patients 1, 2, 3, 4. Only after successful culture establishment mutational hotspots of the BRAF, NRAS, KRAS loci were sequenced. HGIEN = high-grade intra-epithelial neoplasia; M = male, F = female, LN = lymph node, 5-FU = 5-Fuoruracil, wt = wild-type.

When naïve single cells were embedded in Matrigel and cultured in our CSC-medium, a more complex self-organization was observed in all patients analysed. Similar to floating spheroids in low-attachment conditions, tumour cells embedded in Matrigel could not establish a stable organoid culture under FGFR-inhibition (Fig. 1C). In general, we observed that initial spheroid formation in low-attachment culture was more efficient than organoid formation in Matrigel, resulting in more and larger spheroids within the first days after the initiation of cultures. This might be due to cell aggregation, which is reduced by the high viscosity of Matrigel. Within 30 d, however, Matrigel-cultured organoids increased massively in size, composed of more complex structures and proliferated faster than floating spheroids.

Organoids established in CSC-medium were analysed for genetic hotspot mutations of the *BRAF*, *NRAS* and *KRAS* genes by sanger sequencing. We identified activating mutations in all patients except Patient 2. To further confirm the cancer status of the organoids, we analysed their genomes for copy number variations (CNV) by comparative genomic hybridization. We identified highly aberrant genomes in biopsies from Patient 1 and 2, which were derived from metastatic sides and less but significant CNVs in biopsies from primary tumours of patient 3 and 4 (Fig. S1). When we initially established organoid cultures from these chemo-refractory metastases, we observed that the withdrawal of EGF had no effect on culture initiation, which has been described in organoids obtained from progressed colon cancer^3,12^. In our organoid culture model, the non-dependence on exogenous EGF supplementation can be explained by the presence of activating mutations of genes of the RAS family observed in Patients 1, 3 and 4^15^. Patient 2, however, carried a wild-type RAS gene but developed resistance against the EGFR-inhibitor Cetuximab during pre-treatment, indicating constitutively activated EGF signalling (Table 1).

These experiments revealed that highly malignant CSCs, known to be non-dependent on Wnt stimulation, cannot establish organoid or spheroid cultures under active FGFR suppression, thus implying suppressed self-renewal.

### Self-renewal in advanced colorectal cancer organoids

**Figure 2:**
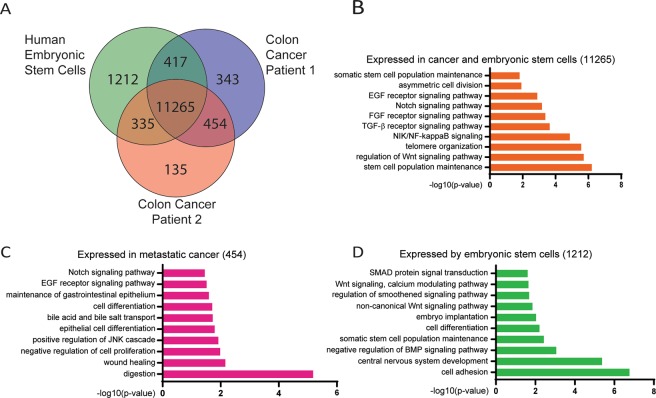
Comparative microarray transcriptome analysis of genes expressed in human embryonic stem cells and organoids derived from Patient 1 and Patient 2. (**A**) Venn diagram showing the number of expressed genes commonly or specifically expressed by each sample. (**B**–**D**) Gene Ontology (GO) terms of significantly enriched gene patterns. Only GOs of biological process with a p-value < 0.05 were considered.

As our CSC-medium was intended to maintain cells in an undifferentiated state, we comparatively analysed the transcriptomes of the two metastatic biopsies and hESCs- a model for FGF2-driven self-renewal (Fig. 2A, Table S3)^6^. We found the majority of expressed genes (11,265) expressed by hESCs and metastasis-derived organoids. 1,212 genes were detected as expressed exclusively in hESCs. Notably, both cancer samples expressed more genes (454) in common than distinctively.

To identify functional annotations and enriched pathways among these expressed genes, we used the Gene Ontology (GO) tool and specifically focused on Biological Processes (BP) associated with stemness or self-renewal^16^. In the set of 11,265 genes expressed in all samples, we detected GO-terms of stem cell-associated attributes such as “*telomere organization*” and “*asymmetric cell division*”. Self-renewal-associated pathways such as Notch, FGF2, TGF-β and Wnt signalling were also enriched in this gene-set. Factors of these pathways, together with other stem cell-related genes for example, *SALL4*, *SOX2* or *CDH2* where associated with the GO term “(*somatic*) *stem cell population maintenance*” (Fig. 2B, Table S4). In addition to those listed, we found several GO-terms associated with oxidative stress, hypoxia, proliferation, DNA repair, apoptosis, inflammation and metabolic changes.

Genes expressed solely by patient-derived organoids displayed an intestinal differentiation pattern next to stem cell circuits shared with hESCs (Fig. 2C, Table S5). The GO-terms “*digestion*”, “*maintenance of gastrointestinal epithelium*” and “*bile acid and bile salt transport*” comprise intestinal specific genes of the trefoil factor (TFF)- or the aldo-keto reductase (AKR)-family. Interestingly, metastasis-derived organoids exerted extended Notch and EGF signalling. Besides genes expressed in common with hESCs, cancer cells expressed Epiregulin (*EREG*) and Amphiregulin (*AREG*) of the EGF-receptor (EGFR) pathway, generally associated with progressed cancer^17,18^.

Genes expressed by hESCs are associated with the GO-term “*cell adhesion*”, for example α-catenin (*CTNNA2*) and cadherin-11 (*CDH11*), are associated with adherens junctions but also fulfil functions in stem cell proliferation (Fig. 2D, Table S6)^19,20^. The pluripotency-associated genes *NANOG*, *LIN28* and *SALL1*, were exclusively expressed by hESCs. In accordance with the mechanism of FGF2 regulated TGF-β signalling, the GO-terms “*negative regulation of BMP signaling pathway*” and “*SMAD protein signal transduction”* were detected in our hESC culture^6^. Most genes associated with this circuit were shared with metastasis-derived organoids indicating that FGF2 also modulates self-renewal associated pathways in our organoid culture.

### FGF signalling inhibits cellular differentiation

**Figure 3:**
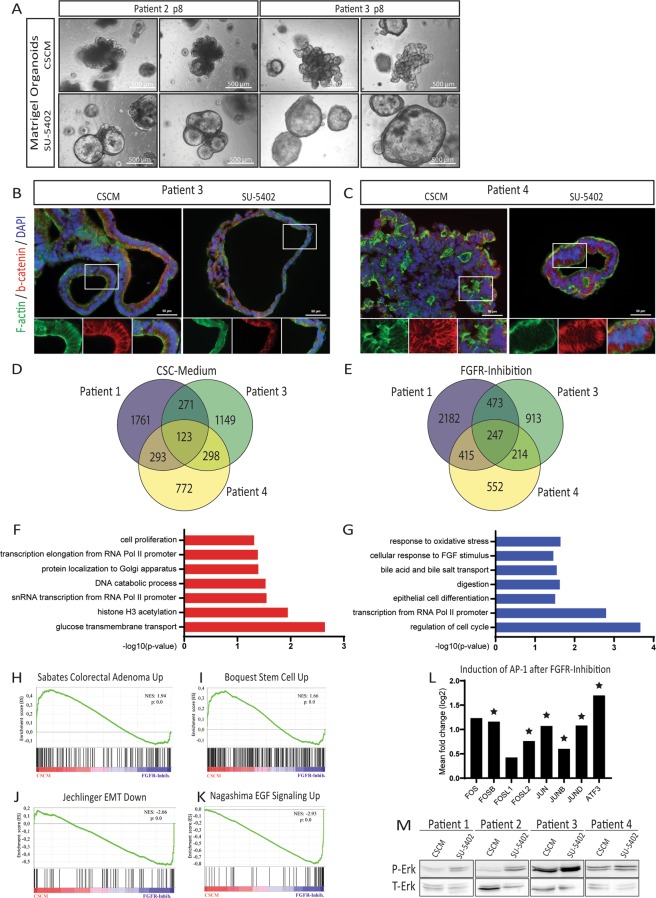
FGFR-inhibition induces cyst formation and cellular differentiation in pre-established organoids. (**A**) Cyst induction by FGFR-inhibition in Matrigel-embedded organoids of p8 after 7 d of SU-5402 treatment. (**B**) Cryo-sections of organoids from patient 3 and (**C**) patient 4, cultured with CSCM or SU-5402, stained for F-actin and β-catenin. (**D**) Venn Diagram of genes differentially higher expressed (p < 0.05) in organoids cultured in CSC-Media or (**E**) after FGFR-inhibition. (**F**) Significantly enriched GO-terms of biological processes of genes higher expressed after CSC-Media culturing or (**G**) higher expressed after FGFR-inhibition. Only genes with p < 0.05 and limma-p < 0.05 were considered for GO term-analysis. (**H**–**K**) Gene-sets significantly over represented in the CSC-media condition or the FGFR-inhibition condition. NES = normalized enrichment score. (**L**) Mean fold change (log2) values of single genes belonging to the AP-1 transcription factor complex of Patient 1, 3 and 4 (n = 3). Differential expression values were obtained by microarray analysis; star symbol indicates significance as limma p-value < 0.05. See Table S9 for log2 ratio of single probes detected. (**M**) Western blot of protein lysates from organoids cultured in CSC-Medium or with SU-5402 (each patient sample, n = 1). Phosphorylated ERK (P-ERK) is compared to total amount of ERK-protein (T-ERK). To increase clarity, blots were cropped. Full-length blots are displayed in Fig. S4.

By comparative transcriptome analyses, we identified numerous self-renewal-associated pathways employed by pluripotent as well as by CSCs in our organoid culture. Specifically, genes linked to FGF2 and TGF-β signalling were shared by these two cell types. We initially showed that SU-5402 treatment abrogates organoid formation by naïve unsorted cells, this would imply impeded self-renewal by FGFR-inhibition. To analyse this effect in depth, we applied SU-5402 treatment on Matrigel-embedded organoids pre-established using our CSC-medium. Interestingly, the effect of FGFR-inhibition was less severe in established organoid cultures (Fig. 3A). Pre-established organoids underwent altered morphologies after 7 d of FGFR-inhibitor treatment but were still able to proliferate and could also be passaged. Interestingly, organoids cultured in CSC-medium usually grew in a densely packed, grape-like or dishevelled structure, and we observed increased cyst formation under FGFR-inhibition. These liquid-filled cysts consisted of a thin layer of epithelial cells with dark patches of apoptotic or necrotic cells shed into the lumen. Cysts also appeared at a low frequency in our standard culture conditions, but their occurrence increased significantly under FGFR-inhibition. This observation was also confirmed by immunofluorescence staining on cryo-sections of Matrigel-embedded organoids (Figs. 3B,C and S2). Tissue polarity in terms of a crypt-lumen axis as in healthy mucosa-derived organoids is usually lost in early adenoma and could not be detected in our organoids as expected^13,21^. In CSC-medium cultured organoids, cell density was higher compared to FGFR-inhibition. Employing F-actin staining, we assessed cellular morphology and found mostly columnar-like shaped cells with a clear epithelial polarity and nuclei located on the basal side of the cells. However, in organoids derived from Patient 3, inhibition of FGF signalling induced mainly smaller cuboidal cells. In Patient 4, a biopsy of a non-metastatic but poorly differentiated tumour, we observed a less pronounced impact on cell morphology, corroborating previous observations in poorly differentiated carcinomas^3^. In the FGFR-inhibitor treated sample, we observed a lower cell density and a clear lumen-like structure. Interestingly, the cells kept their polarized, epithelial structure but were arranged in a stratified order of multiple layers of cells (Fig. 3C).

Wnt signalling is an important driver of stem cell activity and self-renewal. In the activation of the canonical pathway, β-catenin (*CTNNB1*) translocates to the nucleus where it induces gene expression^22^. We analysed the subcellular localization of β-catenin by immunofluorescence staining on sections of organoids cultured with or without FGFR-inhibition. We mainly detected β-catenin localized to the lateral cell membranes. As a component of adherens junctions, β-catenin interacts with E-cadherin (*CDH1*) and α-catenin contributing to cell polarity in epithelial cells. Besides a weaker cytoplasmic staining, the β-catenin signal was always excluded from the nucleus, independent of culture conditions. These results indicate that the morphological change of a complex organoid structure into cysts is independent of β-catenin translocation.

The intestinal differentiation marker Cytokeratin-20 (*KRT20*) was heterogeneously expressed within individual organoids (Fig. S2). In some cysts, KRT20 staining appeared stronger after FGFR-inhibition. This trend, however, could not be significantly quantified. The cystic morphology after FGFR-inhibition might indicate pronounced differentiation of these organoids.

Aiming for a deeper understanding of the underlying mechanisms that confer cyst formation and induce differentiation, we used comparative microarray transcriptome analysis of organoids cultured in our standard FGF2-containing CSC-medium or after FGFR-inhibition by SU-5402 treatment. Venn diagrams depicting numbers of regulated genes expressed for each condition indicated a heterogeneous response between all samples analysed (Fig. 3D,E, Tables S7 and S8). While many genes responded to the modulation by FGF2, only a few were regulated in common in all three samples. In addition to genes associated with glucose or folate metabolism (e.g. *SLC2A12*, *SLC2A14*, *FOLR1*), we identified TGB-β-induced (*TGFBI*) among the highest overrepresented genes in our CSC-medium culture (Table S9), indicating FGF2 downstream effects mediated by TGB-β signalling.

Genes overexpressed in common after FGFR-inhibition correlated with drug metabolism (e.g. *CYP1A1*, *CYP51A1*) as well as with proliferation and differentiation (e.g. *EGR1*, *ATF3*, *FOSB*, *JUN*) (Table S8). GO-term analysis of this gene-set yielded only few significant results due to the low number of commonly regulated genes (Table S10). Most of these GO-terms for the CSC-medium condition were associated with active transcription, cell proliferation or an increased glucose uptake (Fig. 3F). After FGFR-inhibition, we found cell-cycle regulators as well as many genes annotated with “*epithelial cell differentiation”*, “*digestion*” or “*bile acid and bile salt transport*” as enriched GO-terms (Fig. 3G, Table S11).

To additionally include genes with minor but still significant changes, we conducted gene-set enrichment analysis (GSEA) which includes all expressed genes ranked by ratio.

The FGF2-driven induction of several Wnt and TGF-β modulators in CSC-media cultured organoids correlates with gene-sets of colorectal adenomas compared to healthy mucosa or with an adult stem cell gene signature adding additional credence to our hypothesis that stem cell features of these tumour cells are dimished by FGFR-inhibition (Fig. 3H,I, Table S12)^23,24^.

Active TGF-β signalling can induce epithelial-mesenchymal-transition (EMT), a critical process for cancer cell invasiveness and dissemination^8^. Genes directly associated with EMT together with a mesenchymal and self-renewing phenotype (*TGFB3*, *ID1*, *ID2* and *ID4*) were found to be overexpressed in organoids of the CSC-medium condition, while their antagonist, *BMP4* and other epithelial genes such as E-cadherin (*CDH1*), *FOS*, *ATF3*, *EGR1 or DUSP1* were induced by FGFR-inhibition (Fig. 3J)^8,25,26^. Interestingly, these genes belong to the group of immediate-early genes (IEGs) which are usually transiently induced by growth factors such as EGF or FGF2^6,27^. We, however, detected these genes induced after 7 d of FGFR-inhibition, when no growth factors were added to the medium. In addition to the constitutive expression of the IEGs, we found several EGFR target genes up-regulated and, consequently, many EGF signalling-related gene-sets enriched in our gene list of FGFR-inhibitor treated organoids (Fig. 3K, Table S12)^28^. Most prominent was the persistent up-regulation of the subunits of the Activator Protein-1 (AP-1) which is a homo- or heterodimer of proteins of the FOS family (*FOS*, *FOSB*, *FOSL1*, *FOSL2*) and the JUN family (*JUN*, *JUNB*, *JUND*) as well as the additional interaction partner *ATF3* (Fig. 3L)^29^. These genes are associated with active MAPK signalling. Since many MAPK inhibiting dual specificity phosphatases (DUSP) were also upregulated (e.g. *DUSP1*, *DUSP4*, *DUSP5*, *DUSP8*), we analysed the phosphorylation of ERK, the most downstream kinase of the MAPK cascade. An increased ERK-phosphorylation is described in RAS-mutant as well as in chemo-refractory cancers which applies to most our patients (Fig. 3M)^30^. However, we found altered ERK-phosphorylation induced by FGFR-inhibition in all patients thus implying that FGF signalling can modulate MAPK-phosphorylation in RAS-mutant (Patient 1,3,4) as well as in RAS-wild-type tumours (Patient 2).

Besides the described effects detected in all patients, we also found individual differences in each patient (Table S13 and Fig. S3). The strongest correlation of differentiation and increased proliferation under FGFR-inhibition but induced self-renewal under CSC-medium treatment was observed in Patient 1. We found several gene-sets and pathways overrepresented in the FGFR-inhibitor condition not only connected to RAS-induced MAPK activity, but also an enrichment of genes regulated by STAT3, JNK and AKT as well as an increased expression of the proliferation marker *MKI67*. In CSC-medium cultured organoids, on the other hand, we detected an induction of TGF-β and of all four members of the inhibitor-of-differentiation (ID) gene family, which are directly involved in self-renewal of colon cancer stem cells^25^. The most significant induction of genes associated with stem cells was found in Patient 3 in the CSC-medium condition. On the single-gene-level, an up-regulation of *TGFB2* and *INHBA* together with the Wnt-target genes *LGR5*, *TCF4* and *LEF1* was most prominent in organoids derived from this biopsy. In Patient 4, the gene expression pattern was also dominated by a strong RAS-ERK signalling under FGFR-inhibition. Distinct to other patients, however, was a slight downregulation of *TGFB1* during CSC-medium culture as well as a downregulation of *ID1*, *ID2* and *ID3*, but at the same time a reduced expression of the differentiation markers *BMP2* and *KRT20* and a simultaneous up-regulation of the stem cell factor *SOX2*.

These results imply that FGF2 signalling has heterogeneous molecular effects among distinct patients, but also has common effects in the inhibition of cell differentiation, a hallmark of self-renewal.

## Discussion

Currently existing organoid biobanks comprise nearly all kinds of colon cancer subtypes with 60–100% efficiency in the establishment of cultures^2,3,12,31,32^. While most projects focus on primary cancer tissue, metastatic biopsies are rarely analysed^2,33–35^. From many organoid-based studies, it has become clear that niche requirements and, by that, cell culture specifications reduce with tumour progression while the number of putative CSCs increases^1^. However, it is still challenging to culture primary or metastatic colorectal cancer tissue in a way that self-renewal and self-organization are maintained. In some protocols of CSC culture, FGF2 is an important ingredient for maintaining stemness, while the human intestinal stem cell (HISC) medium described by Sato and Clevers uses a complex modulation of the Wnt, ALK/BMP, TGF-β and p38-MAPK pathways by Rspo1, Noggin, A83-01 and SB202190^11,12,31^. Our medium contained only serum replacement supplements as well as EGF and FGF2. Pathways shared by hESCs and metastatic cancers corroborated with our hypothesis that FGF2 regulates self-renewal via TGF-β modulation. While the HISC-medium uses A83-01, an inhibitor of TGFβR1 (*ALK5*), to avoid TGF-β-induced cytostasis, we observed an induced TGF-β expression after FGF2 stimulation without any growth inhibitory effects. This tumour promoting effect of TGF-β has been reported to be restricted to highly mutated and progressed cancers and might further be context dependent^8^.

The fact that other protocols omit FGF2 in their culture media but use, depending on tumour’s stage, other activators of different pathways instead, indicates that self-renewal can be activated via additional pathways which have many interconnections^4^. We however cannot exclude that autocrine FGF signalling is operative in these cultures.

We indeed found differential expression of genes of other well-described pathways such as the NFκB- and the Notch-pathway. In our organoid culture system of highly malignant primary and metastatic tumour cells, which are non-dependent of any exogenous Wnt, Rspo or TGF-β modulation, FGF signalling can act as a master regulator of cancer stem cell self-renewal.

Increased expression of FGFRs as in prostate or in non-small cell lung cancer (NSCLC) is not described in CRC^36,37^. However, in a genome-scale analysis by The Cancer Genome Atlas (TCGA) 26% (51/195) of all patients had genomic or transcriptome alterations, mostly mRNA up-regulation, in at least one of the FGFR genes or its adaptor proteins FRS2/3, thus implying that cancer cells might benefit from an increased FGF signaling^5,38^.

To our best knowledge, this is the first study analysing the effect of FGF2 signalling on CSC-organoid cultures. We used the small molecule SU-5402 for FGFR-inhibition. At the concentration used in this study, SU-5402 is a potent and selective FGFR1 and angiogenesis inhibitor. Other tyrosine kinases, such as PDGFR, are reported to be inhibited only at higher concentrations^14,39^. This active suppression of FGFR downstream effects might have broader consequences than only omitting FGF stimulation. When we inhibited FGFR signalling, EGF was not present in the culture medium. Interestingly, we identified many enriched gene-sets of an induced EGF-pathway after FGFR-inhibition. Similar to observations in patients treated with anti-EGFR therapy, we detected increased RAS signalling and ERK-phosphorylation. In some cancers, for example NSCLC, it has been described that anti-EGFR therapy induced FGFR signaling^37^. In many aspects, we observed the reverse effect of an increased endogenous EGF signalling after FGFR-inhibition.

It has been shown for MAPK signalling that regulation is preserved in RAS-mutant tumours^30,40^. While FGFR-inhibition led to an enhanced MAPK signalling as indicated by AP-1 induction and increased ERK-phosphorylation, we also observed induced differentiation, a loss of most stem cell markers and an epithelial phenotype. This contrasts with results of Blaj *et al*., who reported EMT-induction together with increased expression of stem cell antigens CD44, ASCL2 and EPHB2 upon MAPK stimulation in KRAS wild-type and mutant CRC cells^40^.

As for MAPK signalling in RAS-mutant tumours, a modulation of β-catenin and the Wnt signalling pathway has been described in APC-mutant CRC. Even though we did not add any direct Wnt regulators to our medium, many Wnt-modulating proteins, such as *DKK1/2/4*, *LGR5*, *NOTUM and APCDD1* were regulated by differential FGF signalling. Nuclear translocation of β-catenin, however, was not observed in our organoids. In a study of patients with APC-mutant CRC, nuclear translocation was restricted to the proliferative front of highly invasive cancers^41^. In intestinal organoids of mice with floxed APC alleles (APC^fl/fl^), β-catenin was located to the lateral cell membranes^21^. Control wild-type organoids in this study grew in a highly branched, crypt-like morphology, while APC^fl/fl^-organoids grew as cysts. In this context, cyst-formation indicates a loss of tissue-polarity representing a model of an early and well differentiated adenoma. Grape-like organoids with high cell density can be observed only in more advanced cancers with acquired mutations^3,21^. In our study, cyst-formation increased extensively under FGFR-inhibition and accordingly, transcriptional changes indicated a differentiated phenotype. It can be assumed that the heterogeneity of cells within cysts after FGFR-inhibition decreased whilst the RAS-mutated phenotype became dominant and resulting in increased proliferation as, manifested by increased expression of the proliferation marker- *MKI67*.

With ongoing culturing, cells adapt to culture conditions which might be accompanied with a loss of heterogeneity and with an increased robustness against unfavourable culture conditions. This might explain why FGFR-inhibition led to a complete suppression of organoid formation from naïve cells, while established organoids changed their morphology but kept, or even increased, their ability to proliferate. Additionally, the presence of cell-cell-contact in already established organoids allows juxtacrine and paracrine signalling, which might increase the viability and proliferation of the cells.

Selection of highly proliferative clones and, by that, neglecting quiet or dormant stem cells, is a drawback of many cell culture models and might apply for organoid cultures as well. By demonstrating a subpopulation of CSCs being sensitive to FGF-modulation, we highlight one of many aspects of CSCs great heterogeneity, maybe the biggest challenge in cancer research and its treatment.

## Material and Methods

### Tumour dissociation and culture initiation

Surgically resected tumour material of nine consecutive unselected CRC patients (UICC stages I-IV) who gave written informed consent were included in this study. The study was approved by the institutional ethics committee of the University Hospital Düsseldorf, Germany (#4664). We hereby confirm that all research was performed in accordance with relevant guidelines and regulations and informed consent was obtained from all participants.

Tissue was washed in PBS including antibiotics/antimycotics (200 U/ml Penicillin, 200 µg/ml Streptomycin, 500 µg/ml Gentamicin, 25 µg/ml Amphotericin B). Tumour fragments were minced using scissors and scalpel until small pieces of maximal size of 0.5 mm^3^ were obtained. Smaller pieces were incubated in digestion buffer consisting of Advanced DMEM/F12, 10 mM Hepes, 1% GlutaMAX, 2% B-27 media supplement without retinoic acid, 1% N-2 media supplement, 2 µg/ml Heparin, 1 mM N-Acetylcystein, 50 ng/ml human EGF, 50 ng/ml human FGF2, 10 µM Y-27632, 1 mg/ml Collagenase IV, 20 µg/ml Hyaluronidase IV and antibiotics/antimycotics under constant rotation for 2 h at 37 °C with occasionally pipetting up and down^11,42^.

FBS was added up to 10% to stop the reaction. After centrifugation (300 × g, 5 Min) the supernatant was discarded, and cell suspension was washed in 30 ml PBS and filtered through a 70 µm and a 30 µm nylon mesh and centrifuged (300 × g, 5 Min) afterwards. The cell pellet was resuspended in Cancer Stem Cell Medium consisting of Advanced DMEM/F12, 10 mM Hepes, 1% GlutaMAX, 2% B-27 media supplement without retinoic acid, 1% N-2 media supplement, 2 µg/ml Heparin, 1 mM N-Acetylcystein, 50 ng/ml human EGF, 50 ng/ml human FGF2, 10 µM Y-27632 and antibiotics/antimycotics.

Cells were cultured in ultra-low attachment wells (96 Well, 5,000 cells per well in 250 µl media) or as organoids in growth-factor-reduced Matrigel (Corning, phenol red-free) droplets (5,000 cells per 50 µl Matrigel droplet). Matrigel droplets were covered with 500 µl medium. Medium was changed every other day. After 48 h of culturing, Y-27632 was omitted from the media and antibiotics/antimycotics treatment was reduced to 100 U/ml Penicillin and 100 µg/ml Streptomycin. For FGFR-inhibition experiments, 10 µM SU-5402 (Sigma) was added to the medium. SU-5402 powder was solved in DMSO (30 mM) according to the manufacturer’s protocol and was diluted 1:3,000 for a working concentration. The same DMSO dilution was equally added to control experiments.

### DNA isolation

Matrigel cultured organoids were treated with digestion buffer for 30–60 Min at 37 °C to remove remaining Matrigel. For DNA isolation, the DNeasy Blood & Tissue Kit (Qiagen) was used. According to the manufacturer’s protocol, organoids were lysed in Buffer AL with proteinase K and DNA was isolated using silica-based columns.

### Analyses of the mutational status of BRAF, KRAS and NRAS using Sanger sequencing

DNA amplification of selected regions from BRAF (Exon 15), KRAS (Exon 2–4) and NRAS (Exon 2–4) was performed using specific primers (Table S1). Amplification was controlled using agarose gel electrophoresis and amplified fragments were subjected to direct Sanger sequencing from either one end or both ends at our core facility (BMFZ, Düsseldorf) using amplification primers as indicated (Table S1). Resulting DNA sequences were analysed for nucleotide variants using Chromas software (Technelysium), BLAST alignment tool and UCSC genome browser^43,44^.

### RNA isolation

For RNA isolation, Matrigel-cultured organoids were treated with digestion buffer for 30–60 Min at 37 °C to remove remaining Matrigel. After washing in PBS, organoids were lysed in 500 µL TRIzol RNA isolation employed the Direct-zol RNA Isolation Kit (Zymo Research) according to the manufacturer’s protocol including DNase treatment.

### Protein isolation and western blotting

After removing remaining Matrigel using digestion buffer, organoids were lysed in ice-cold RIPA buffer with proteinase-inhibitor (1:7) and Phosphatase-Inhibitor (1:10) using a Hamilton syringe. The solution was centrifuged at 4 °C, 10,000 × g for 10 Min and the supernatant was taken for BCA (Pierce BCA Protein Assay Kit, Thermo Scientific) protein concentration measurement.

40 µg of protein were analysed by Western blot using antibodies for total-ERK (CST) and phospho-ERK (CST) (Table S2). Equal loading of protein was assessed by Ponceau staining after transfer. For signal detection HRP-coupled secondary antibodies were used followed by chemiluminescence detection (Amersham ECL Prime (GE Healthcare), Fusion FX instrument, (PeqLab)).

### Organoid embedding and Cryo-sections

Organoids were embedded in OCT by following published protocols with some modifications^45^. Matrigel droplets containing cultured organoids were treated with digestion buffer to remove Matrigel. Free floating organoids were fixed in 4% PFA for 15 Min at RT. Fixed organoids were incubated in 10% sucrose solution, followed by 20% and 30% sucrose solution, each for 30–60 Min for dehydration. To later re-identify the organoids, an alcian-blue staining was done by adding alcian blue (1% in 3% acetic acid) solution to the 20% sucrose solution (1:100 dilution). Organoids were placed in an appropriate mould and left to settle down. All remaining sucrose solution was carefully removed. The mould was filled with OCT followed by mild agitation for 15 Min to collect all organoids in the centre of the mould. Embedded organoids were frozen at −80 °C. 10 µm sections were done using a cryostat with a cutting temperature of −21 °C.

### Immunocytochemistry

Slides were washed in PBS to remove all remaining OCT. All steps were carried out in a wet chamber. Unspecific binding was blocked by incubation for 2 h at room temperature (RT) with blocking buffer (PBS, 10% normal donkey serum, 1% BSA). For all intracellular targets, cells were permeabilized by including 0.5% Triton and 0.05% Tween in all blocking, washing and incubation steps.

Primary antibodies for Cytokeratin-20 (abcam) and β-catenin (CST) were diluted in blocking buffer and incubated overnight at 4 °C (Table S2). After primary staining, slides were washed three times in PBS. Secondary staining was done at RT for 2 h. For actin filament staining, Alexa Fluor-488 Phalloidin (Invitrogen) was added to the secondary staining following the manufacturer’s protocol. Slides were washed three times and covered with coverslips using DAPI Fluoromount mounting solution. Images were captured using a LSM700 fluorescence microscope

### Microarray transcriptome analysis

Microarray transcriptome profiling was performed at the core facility BMFZ, Düsseldorf. Affymetrix PrimeView Human Gene Chips were used. Bioinformatic analyses were conducted as previously^46^. Details are given in the Supplementary Methods section.

### Comparative genomic hybridization with oligonucleotide microarrays (aCGH)

ACGH was carried out using the Kit Oligonucleotide Array-Based CGH for Genomic DNA Analysis, Version 7.5, June 2016 (Agilent Technologies). Genomic DNA (gDNA) isolation was conducted as described above. Sample preparation, labelling and microarray processing was performed following the manufacturer’s instructions and as described before^47^. Details are given in Supplementary Methods.

## Supplementary information

Supplementary Methods, Table S1-2, Figure S1-4

Supplementary Dataset 1

## Data Availability

The datasets pertaining to the current study are available from the corresponding author upon request.
