# Supplementary material for: FGF Signalling in the Self-Renewal of Colon Cancer Organoids
Source: Sci Rep. 2019 Nov 22;9:17365. doi: 10.1038/s41598-019-53907-7 (PMC6874569; doi:10.1038/s41598-019-53907-7)
Supplement: Supplementary file 1 — Supplementary Methods, Table S1-2, Figure S1-4 [file 41598_2019_53907_MOESM1_ESM.docx]

**FGF Signalling in the Self-Renewal of Colon Cancer Organoids**

Jörg Otte^1^, Levent Dizdar^2^, Bianca Behrens^2^, Wolfgang Goering^3^, Wolfram T. Knoefel^2^, Wasco Wruck^1^, Nikolas H. Stoecklein^2#^ and James Adjaye^1#^*

#contributed equally

*corresponding author

^1^Institute for Stem Cell Research and Regenerative Medicine, University Hospital and Medical Faculty of the Heinrich-Heine University Düsseldorf, Düsseldorf, Germany.

^2^General, Visceral and Paediatric Surgery, University Hospital and Medical Faculty of the Heinrich-Heine University Düsseldorf, Düsseldorf, Germany.

^3^Institute for Pathology, University Hospital and Medical Faculty of the Heinrich-Heine University Düsseldorf, Düsseldorf, Germany.

**Supplementary Methods**

**Microarray transcriptome analysis**

As described before, gene expression was determined using a detection p-value <0.05^1^. Transcriptome data of the hESC lines H1 and H9 were obtained and described before^2^. Mean expression of H1 and H9 was calculated and used for comparative analyses. Expressed genes in different cell types or under different conditions were compared by Venn diagrams using the R package VennDiagram^3^. The limma Bioconductor package and the p-value adjustment method from the q-value package were used to assess differential expression^4,5^. Genes were graded as differentially expressed for limma p-value <0.05. Genes were considered as upregulated with ratio >1.33, or as downregulated with ratio < 0.75.

Gene-sets of commonly or individually expressed genes by different cell types, or significantly up- or downregulated after SU-5402 treatment were further analysed for Gene Ontology terms of biological processes (GO-BP) using the DAVID web tool (https://david.ncifcrf.gov/)^6^.

Gene-set enrichment analysis (GSEA) was performed with the GSEA java application downloaded from Broad institute for the comparison of all treated patients together versus all controls together and on the level of individual treated versus untreated patients. For the joint comparison n=1000 permutations and default parameters were used, for the individual patient comparisons also n=1000 permutations and default parameters were used except for the metric “Diff of classes” and the permutation over the “gene_set“ ^7^.

**Comparative Genomic Hybridization with Oligonucleotide Microarrays (aCGH) and data analysis**

As describe before, 1 µg of DNA was used for restriction digestion of gDNA using restriction enzymes RsaI and AluI to obtain fragments of 200-500 bp^8^. Digested gDNA was random-primed labelled with Cyanine-5 or Cyanine-3-dUTP and purified afterwards. After determining sufficient labelling efficiency, samples were hybridized to the 4x180k platform. For evaluation, the Microarray Scanner G2565CA (Agilent Technologies) was used (3mm resolution, 16-bit colour depth). A gender-mismatched reference DNA was used as an internal technical control for each sample. Aberration calling was determined by the HaarSeq-based algorithm using the following filter thresholds: $\pm$3 oligos and $\pm$0.2 log2ratio^9^.

**Table S1.**

Oligonucleotides for BRAF, KRAS and NRAS amplification or sequencing, respectively.

| **Gene** | **Exon** | **Name** | **Seq (5‘à3‘)** | **Used for sequencing** |
| --- | --- | --- | --- | --- |
| BRAF | 15 | BRAF_15_for | TGCTTGCTCTGATAGGAAAATG | Yes |
| BRAF | 15 | BRAF_15_rev | AGCCTCAATTCTTACCATCCA | No |
| KRAS | 2 | KRAS_2_for | AGGCCTGCTGAAAATGACTGAA | No |
| KRAS | 2 | KRAS_2_rev | AAAGAATGGTCCTGCACCAG | Yes |
| KRAS | 3 | KRAS_3_for | GGATTCCTACAGGAAGCAAGT | No |
| KRAS | 3 | KRAS_3_rev | TGGCAAATACACAAAGAAAGC | Yes |
| KRAS | 4 | KRAS_4_for | GGACTCTGAAGATGTACCTATGG | Yes |
| KRAS | 4 | KRAS_4_rev | TCAGTGTTACTTACCTGTCTTGT | Yes |
| NRAS | 2 | NRAS_2_for | ACAGGTTCTTGCTGGTGTGA | No |
| NRAS | 2 | NRAS_2_rev | CACTGGGCCTCACCTCTATG | Yes |
| NRAS | 3 | NRAS_3_for | GTGGTTATAGATGGTGAAACCTGT | No |
| NRAS | 3 | NRAS_3_rev | TGGCAAATACACAGAGGAAGC | Yes |
| NRAS | 4 | NRAS_4_for | TTCCCGTTTTTAGGGAGCAGA | Yes |
| NRAS | 4 | NRAS_4_rev | TGCAAACTCTTGCACAAATGC | Yes |

**Table S2.**

Antibodies used for Western Blotting and Immunocytochemistry (mAB: monoclonal antibody, pAB: polyclonal AB)

| **Target** | **Host** | **Vendor, Catalog number** | **Dilution** |
| --- | --- | --- | --- |
| Total ERK, p44/42 MAP Kinase | mouse mAB | CST, #4696 | 1:1,000 |
| Phospho-ERK, p44/42 (Thr202/Tyr204) | rabbit mAB | CST, #4370 | 1:1,000 |
| anti-rabbit IgG, HRP-linked | goat | CST, #7074 | 1:2,000 |
| anti-mouse IgG, HRP-linked | goat | abcam, ab6789 | 1:10,000 |
| Cytokeratin-20 | mouse mAB | abcam, ab854 | 1:200 |
| β-catenin | rabbit pAB | CST, #9562 | 1:200 |

**
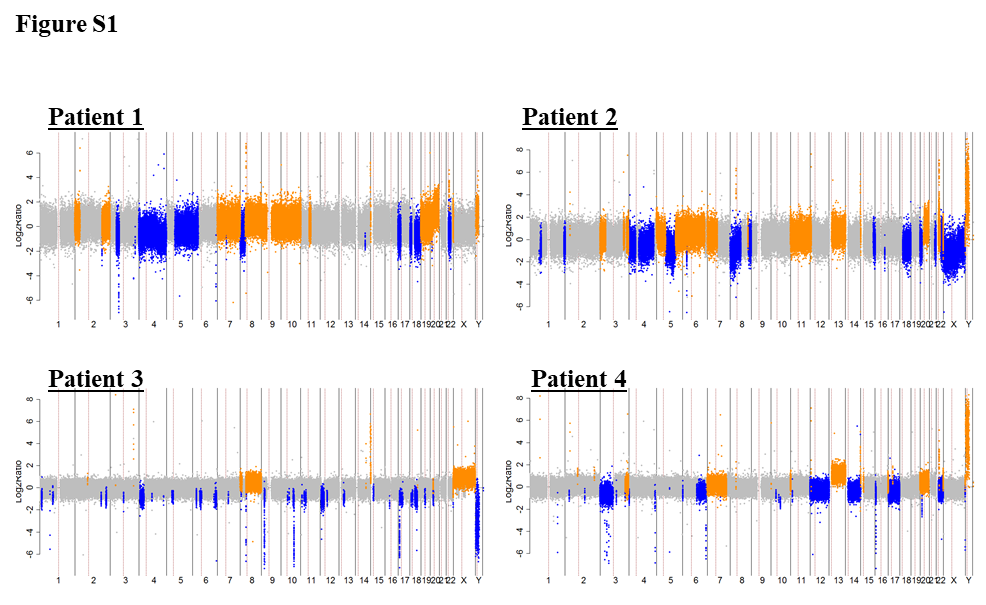
**

Call plot of copy number variations (CNV) after comparative genome hybridization (aCGH). The HaarSeg-based algorithm ($\pm$3 oligos and $\pm$0.2 log2ratio) was used. Orange represents a gain, blue a loss of genomic DNA. As an internal control, a gender-mismatched reference DNA was used.

**
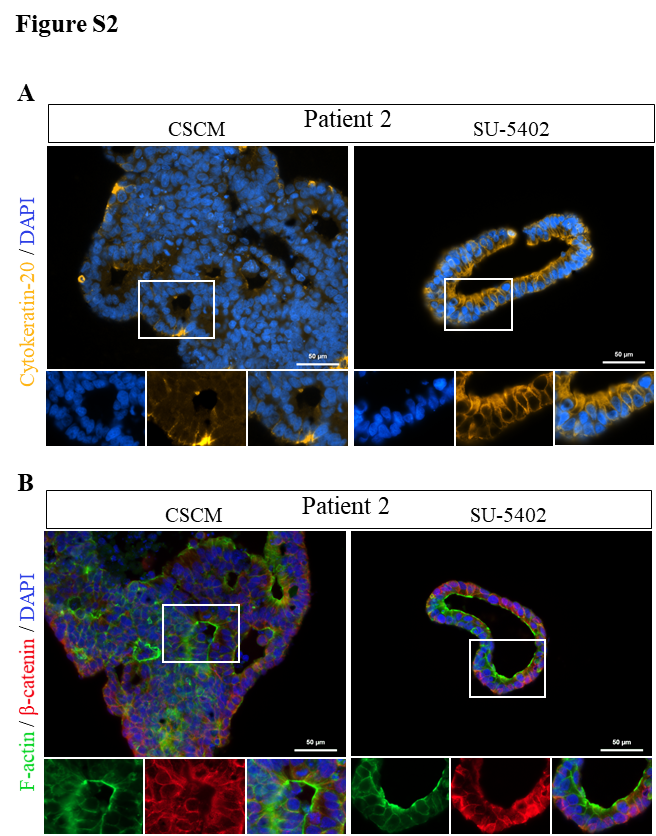
**

Cryo-sections of organoids cultured with CSCM or SU-5402 indicating cyst formation after FGFR-inhibition SU-5402, **(A)** stained for Cytokeratin 20 or **(B)** for F-actin and β-catenin.


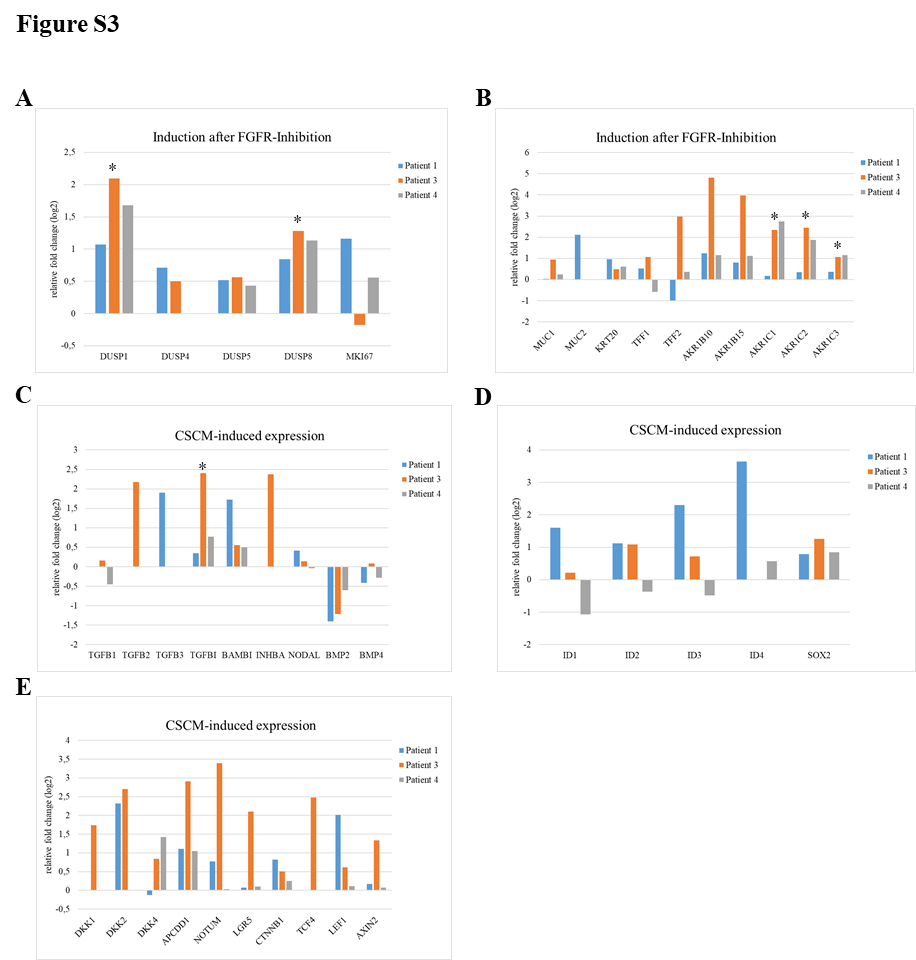


Differential expression of single genes in Patient 1,3 and 4. Expression values were obtained by microarray transcriptome analysis. For each gene in a single patient, the detection p-value is <0.05. * indicates significant regulation among all samples analysed (limma p-value <0.05, see Table S9). Relative expression compared to SU5402 FGFR-inhibition (A, B) or to CSCM treatment (C, D, E) is shown as indicated. **(A)** Several genes of dual specificity protein phosphatases (DUSP) as well as the proliferation marker MKI67 were induced by FGFR-inhibition. **(B)** Colon-specific differentiation markers were induced by FGFR-inhibition. **(C)** In comparison to FGFR-inhibition, the TGF-β pathway was heterogeneously induced by CSCM-treatment. **(D)** Self-renewal associated genes were stronger expressed in CSCM-treated organoids of Patient 1 and Patient 3 compared to FGFR-inhibition. **(E)** Several Wnt modulating factors were upregulated by CSCM-treatment. The intestinal stem cell factors LGR5 and AXIN2 were induced in Patient 3.


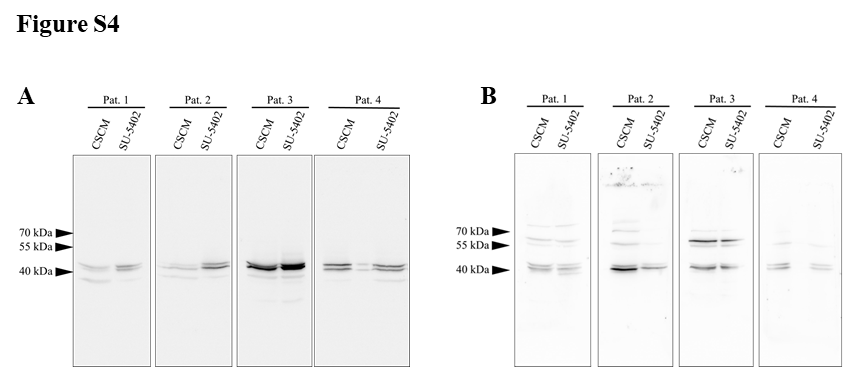


Full-length Western blot of protein lysates from organoids cultured in CSC-Media or with SU-5402. **(A)** Phosphorylated ERK (P-ERK) and **(B)** total amount of ERK-protein (T-ERK). Referring to Figure 3 M.

**Supplementary References**

1 Graffmann, N. *et al.* Modeling Nonalcoholic Fatty Liver Disease with Human Pluripotent Stem Cell-Derived Immature Hepatocyte-Like Cells Reveals Activation of PLIN2 and Confirms Regulatory Functions of Peroxisome Proliferator-Activated Receptor Alpha. *Stem Cells Dev* **25**, 1119-1133, doi:10.1089/scd.2015.0383 (2016).

2 Bohndorf, M. *et al.* Derivation and characterization of integration-free iPSC line ISRM-UM51 derived from SIX2-positive renal cells isolated from urine of an African male expressing the CYP2D6 *4/*17 variant which confers intermediate drug metabolizing activity. *Stem Cell Res* **25**, 18-21, doi:10.1016/j.scr.2017.10.004 (2017).

3 Chen, H. & Boutros, P. M. VennDiagram: a package for the generation of highly-customizable Venn and Euler diagrams in R. *BMC Bioinformatics* **12** (2011).

4 Smyth, G. K. Linear models and empirical bayes methods for assessing differential expression in microarray experiments. *Stat Appl Genet Mol Biol* **3** (2004).

5 Storey, J. D. The positive false discovery rate: a bayesian interpretation and the q-value. *The Annals of Statistics* **31**, 2013-2035 (2003).

6 Huang da, W., Sherman, B. T. & Lempicki, R. A. Systematic and integrative analysis of large gene lists using DAVID bioinformatics resources. *Nat Protoc* **4**, 44-57, doi:10.1038/nprot.2008.211 (2009).

7 Subramanian, A. *et al.* Gene set enrichment analysis: a knowledge-based approach for interpreting genome-wide expression profiles. *Proc Natl Acad Sci U S A* **102**, 15545-15550, doi:10.1073/pnas.0506580102 (2005).

8 Mohlendick, B. *et al.* A robust method to analyze copy number alterations of less than 100 kb in single cells using oligonucleotide array CGH. *PLoS One* **8**, e67031, doi:10.1371/journal.pone.0067031 (2013).

9 Ben-Yaacov, E. & Eldar, Y. C. A fast and flexible method for the segmentation of aCGH data. *Bioinformatics* **24**, i139-145, doi:10.1093/bioinformatics/btn272 (2008).
